# Cross‐species transmission of retroviruses among domestic and wild felids in human‐occupied landscapes in Chile

**DOI:** 10.1111/eva.13181

**Published:** 2021-01-27

**Authors:** Irene Sacristán, Francisca Acuña, Emilio Aguilar, Sebastián García, María José López, Javier Cabello, Ezequiel Hidalgo‐Hermoso, Jim Sanderson, Karen A. Terio, Vanessa Barrs, Julia Beatty, Warren E. Johnson, Javier Millán, Elie Poulin, Constanza Napolitano

**Affiliations:** ^1^ Universidad Andres Bello Santiago Chile; ^2^ Universidad de Chile Santiago Chile; ^3^ Universidad San Sebastián Puerto Montt Chile; ^4^ Parque Zoológico Buin Zoo Buin Chile; ^5^ Global Wildlife Conservation Austin Texas USA; ^6^ University of Illinois Brookfield Illinois USA; ^7^ University of Sydney Sydney New South Wales Australia; ^8^ Department of Infectious Diseases and Public Health City University of Hong Kong Kowloon Hong Kong; ^9^ Smithsonian Conservation Biology Institute National Zoological Park Washinton District of Columbia USA; ^10^ The Walter Reed Army Institute of Research Silver Spring Maryland USA; ^11^ Instituto Agroalimentario de Aragón‐IA2 University of Zaragoza‐CITA Zaragoza Spain; ^12^ Fundación ARAID Zaragoza Spain; ^13^ Instituto de Ecología y Biodiversidad (IEB) Santiago Chile; ^14^ Departamento de Ciencias Biológicas y Biodiversidad Universidad de Los Lagos Osorno Chile; ^15^ Present address: The Walter Reed Biosystematics Unit Smithsonian Institution Suitland Maryland USA

**Keywords:** anthropization, cross‐species pathogen transmission, domestic cat, feline retrovirus, human‐occupied landscapes, *Leopardus guigna*, South America

## Abstract

Human transformation of natural habitats facilitates pathogen transmission between domestic and wild species. The guigna (*Leopardus guigna*), a small felid found in Chile, has experienced habitat loss and an increased probability of contact with domestic cats. Here, we describe the interspecific transmission of feline leukemia virus (FeLV) and feline immunodeficiency virus (FIV) between domestic cats and guignas and assess its correlation with human landscape perturbation. Blood and tissue samples from 102 free‐ranging guignas and 262 domestic cats were collected and analyzed by PCR and sequencing. Guigna and domestic cat FeLV and FIV prevalence were very similar. Phylogenetic analysis showed guigna FeLV and FIV sequences are positioned within worldwide domestic cat virus clades with high nucleotide similarity. Guigna FeLV infection was significantly associated with fragmented landscapes with resident domestic cats. There was little evidence of clinical signs of disease in guignas. Our results contribute to the understanding of the implications of landscape perturbation and emerging diseases.

## 1 INTRODUCTION

Emerging infectious diseases are a serious threat to global biodiversity (Smith et al., 2006). Human‐induced land‐use and ecological changes of natural habitats have been proposed to be major drivers of pathogen emergence (Daszak et al., 2001; Dobson & Foufopoulos, 2001; Murray & Daszak, 2013). There are numerous examples of how anthropized landscapes can facilitate pathogen transfer among domestic animals and wildlife (Foley et al., 2013; Millán et al., 2016; Murray & Daszak, 2013; Riley et al., 2004).

RNA viruses that are transmitted through direct contact are the most‐frequently cited to spread by interspecific host jumping (Woolhouse et al., 2005). They often display high rates of nucleotide substitution and thus have higher capacity to adapt to new hosts (Jones et al., 2008). Feline leukemia virus (FeLV) and feline immunodeficiency virus (FIV) are RNA viruses that cause two of the most common infectious diseases affecting domestic cats (Luria et al., 2004; O’Brien et al., 2012). Both of these felid‐specific retroviruses are horizontally transmitted through saliva or other body fluids (Munro et al., 2014). Transmission occurs mostly through fighting, especially in male animals as they are more aggressive, but also by grooming and sharing of food. Vertical transmission occurs occasionally (Pan et al., 2018). Viremia is persistent in approximately one third of FeLV‐exposed cats; it results in severe clinical disease including immunosuppression, anemia and/or neoplasia (Mullins & Hoover, 1990). The main consequence of FIV in the infected organism is immunosuppression, but it can also produce neoplasia, blood dyscrasias due to myelosuppression and neurological disorders (Hartmann, 2012).

Both FeLV and FIV have worldwide distribution, with prevalence in domestic cats ranging from 3%–30% for FeLV and 0%–50% for FIV (Gleich et al., 2009). The spatial and temporal dynamics of these pathogens differed regionally in the United States, with higher FIV prevalence observed in the southern and eastern U.S., in contrast to higher prevalence of FeLV infections in the western U.S. (Chhetri et al., 2013). This pattern suggests that spatial risk factors can vary geographically, perhaps in response to variations in specific virus strains or rates of vaccination. However, there was no evidence of changes in positivity rates in FIV or FeLV during the studied period (Chhetri et al., 2013).

Infections by FeLV and FIV have also been reported during the last two decades in free‐ranging wild species of the family Felidae worldwide: (lions [*Panthera leo*], Antunes et al., 2008; Florida panthers [*Puma concolor coryi*], Brown et al., 2008; Iberian lynx [*Lynx pardinus*], Meli et al., 2010 and the Tsushima cat [*Felis bengalensis euptilura*], Nishimura et al., 1999; Troyer et al., 2005).

Most FeLV infections in non‐domestic felids appear to be self‐limiting (Sleeman et al., 2001). However, outbreaks linked to mortality have been reported (Iberian lynx; Meli et al., 2009), suggesting that infection can be pathogenic. Possible interspecific transmission of FIV between domestic and wild felids has been described (Carpenter et al., 1996; Mora et al., 2015; Nishimura et al., 1999; Troyer et al., 2005).

Environmental and behavioral barriers may hinder interspecific FIV and FeLV transmission under natural conditions (Troyer et al., 2008). However, human landscape perturbation may facilitate interspecies transmission by increasing contact probability between domestic cats and wild felids. Deforestation and human encroachment into wild habitats are increasing in Chile (Echeverria et al., 2008; Wilson et al., 2005). These perturbations can have a major impact on wild species adapted to the dense temperate rainforests of southern South America, such as the guigna (Sanderson et al., 2002). Guignas have a restricted distribution in central and southern Chile (30°‐48°S) and southwestern Argentina (39°‐46°S west of 70°W; Napolitano et al., 2014). Estimates of plausible lower and upper bounds for the total number of mature individuals range from 6000 to 92,000. Four of the six geographic groups were estimated to have at least 1000 mature individuals (lower bound; Napolitano, Gálvez, et al., 2015). Habitat loss and fragmentation have restricted them to forest fragments surrounded by a human matrix with domestic animals (Gálvez et al., 2013; Sanderson et al., 2002). Although guignas can adapt to perturbed landscapes by using vegetation corridors, increased fragmentation has been associated with wider dispersal and reduced levels of neutral genetic diversity (Napolitano, Díaz, et al., 2015), followed by increased probabilities of encountering domestic carnivores and cross‐species pathogen transmission. Decreased adaptive genetic diversity may also increase susceptibility to infectious diseases, resulting in epidemics, high mortality and the potential of local extinctions (Iberian lynx [*L. pardinus*], Meli et al., 2010; lion (*P. leo*) Roelke‐Parker et al., 1996). Guignas are solitary, pairing only when mating (Sanderson et al., 2002), and thus may be less likely to sustain new infectious agents in their populations. Therefore, sympatric carnivores serving as reservoir hosts may help maintain a relatively high pathogen population, increasing transmission and disease risks (Millán et al., 2009). Guignas are currently classified by the IUCN Red List as Vulnerable (Napolitano, Gálvez, et al., 2015).

Based on the above information, we conducted an extensive molecular survey for FeLV and FIV infection in guignas across their entire distribution range in Chile and in sympatric populations of rural domestic cats. We hypothesized that human landscape perturbation facilitates interspecies transmission of FeLV and FIV, in part through spillover between guignas and domestic cats. Therefore, we expected to find (i) higher FeLV and FIV prevalence in guignas inhabiting human‐dominated landscapes compared to pristine continuous forest habitats, and (ii) high genetic similarity (and shared genotypes) among domestic cat and guigna FeLV and FIV sequences. We also evaluated physiological and pathological patterns in guignas to assess whether FeLV and FIV are causing disease in this threatened species.

## 2 MATERIALS AND METHODS

### 2.1 Study area

**FIGURE 1:**
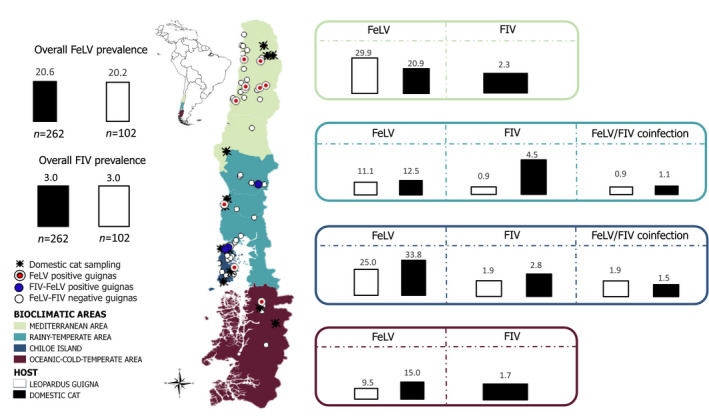
Map of study area showing the overall prevalence of feline leukemia virus and feline immunodeficiency virus in guignas (white color) and domestic cats (shaded black) as well as percent relative prevalence of both viruses in guignas and domestic cats from the different study areas (prevalence is expressed with respect to the sample sizes recorded in each study area)

The study area included four bioclimatic regions in central and southern Chile (33°S–46°S) encompassing the entire current distribution range of the guigna in Chile (Mediterranean area, rainy‐temperate area, Chiloé Island, and oceanic cold‐temperate area; Napolitano, Gálvez, et al., 2015; Figure 1). Study sites included a gradient of different landscape types, ranging from continuous pristine native forest with no human presence to human‐dominated landscapes with high human density and fragments of remnant forest surrounded by a matrix of agriculture, livestock activities and domestic cats and dogs.

### 2.2 Sample collection

Between 2008 and 2018, 102 free‐ranging guignas were sampled using either tomahawk‐like live traps (*n* = 52) or after being received at wildlife rescue and rehabilitation centers (WRRC; *n* = 8). Whole blood samples were collected from these 60 animals, and buffy coats were extracted. We also sampled 42 animals that had been road‐killed or euthanized at WRRC. Bone marrow, Peyer's patches and small intestine samples were collected from these during complete necropsies. Portions of these tissues were archived and subsamples obtained. All the tissues and organs mentioned are target tissues for FeLV and FIV. All live captures and tissue collection were conducted using established methods (Napolitano, Díaz, et al., 2015) following bioethical and animal welfare guidelines and protocols and with prior permission from the Chilean Agriculture and Livestock Service (SAG) and relevant Animal Ethics Committees.

For each guigna, sex, age range (estimated from dentition), physical condition, and clinical signs of disease (Crowe, 1975) were assessed by a veterinarian; date and GPS location of collection/capture were recorded for each animal. A total of 38 females and 64 males, 63 adults and 16 juveniles were sampled (ages of 23 animals were unknown).

We collected whole blood samples from 254 free‐roaming, non‐feral “outside” domestic cats inhabiting rural communities throughout the distribution of the guigna in Chile; buffy coats of each sample were extracted. In addition, bone marrow, Peyer's patches, and small intestine samples were collected during 8 complete necropsies from animals found dead (roadkills) or euthanized at veterinary clinics. Samples were stored frozen at −20°C until PCR analysis. The sex, age class, date, and location were recorded for each individual. A total of 129 females and 133 males, 226 adults, and 36 juveniles were sampled, none of which had been previously vaccinated or neutered.

### 2.3 Genetic analysis

Genomic DNA was extracted using a commercial kit (DNeasy Blood & Tissue kit; Qiagen^®^). DNA amplification was performed by nested PCR in a BIO‐RAD T100 thermal cycler. We conducted MHC class II gene PCR as previously described (Castro‐Prieto et al., 2010) as an internal control to confirm quality of extracted DNA. All samples were validated and subsequently analyzed for FeLV and FIV. We amplified a 291‐base‐pair (bp) fragment of proviral DNA from the FIV *gag* genomic region and a 211‐bp fragment from the FeLV U3 LTR genomic region (unique region which distinguishes endogenous from exogenous FeLV; Miyazawa & Jarrett, 1997) using external and internal primers and conditions described in Mora et al. (2015). All positive samples were tested to amplify longer fragments for FeLV, a 700‐bp fragment of the FeLV U3 region (Miyazawa & Jarrett, 1997) and a 468‐bp segment of the U3 region of the LTR of FeLV‐A (Cattori et al., 2006).

PCR of domestic cat samples and guigna samples was run separately to avoid cross contamination. Ultrapure water was used as a negative control. Positive controls were FIV and FeLV proviral DNA from domestic cats previously diagnosed by PCR and confirmed by nucleotide sequencing. Positive controls were checked against sequences obtained to rule out contamination products and sequences from study animals. All sequences distinct from the positive controls were included in phylogenetic analysis and sequence comparisons. PCR products were separated by electrophoresis in 2% agarose gels stained with SYPRO red protein gel, visualized with a transilluminator. Forward and reverse strands were sequenced by the Sanger method at Macrogen Inc.

Multiple sequence alignments were conducted using the CLUSTAL W algorithm (Geneious**®**). Phylogenetic trees were constructed based on Bayesian and maximum‐likelihood methods. The best model of evolution was selected with jModelTest2 (version 2.1.6; Darriba et al., 2012), under the Akaike Information Criterion. Bayesian inference analysis was performed with Mr. Bayes 3.1.2 (Ronquist & Huelsenbeck, 2003). Four Markov chain Monte Carlo simulations were run for 10^9^ generations (sampling frequency every 1000 generations, burn‐in 25%). The maximum‐likelihood analysis was conducted with RaXML software version 1.5 (Stamatakis et al., 2008; data set resampled 1000 times for bootstrap values). Divergence between guigna and domestic cat FeLV and FIV sequences was calculated with DnaSP v5 (Librado & Rozas, 2009).

A ntST network was generated using the median joining method implemented in popart (Leigh & Bryant, 2015). Phylogeographic structure of FeLV and FIV in guignas and domestic cats was assessed comparing *G*_ST_ and *N*_ST_ coefficients in Permut (Pons & Petit, 1995, 1996; 1000 permutations of *N*_ST_). Genetic structure in guigna and domestic cat host species was estimated using pairwise Phi_st_ tests in Arlequin (Excoffier & Lischer, 2010; 1000 permutations) and the nearest‐neighbor statistic *S*_nn_ in DnaSP v5 (Librado & Rozas, 2009). Newly identified FeLV and FIV sequences were submitted to the GenBank database under the accession numbers indicated in Table S1.

### 2.4 Landscape analyses

To describe landscape features associated with FeLV and FIV infection in guigna, for each sample location we generated a circular buffer area with QuantumGIS 2.14^®^, corresponding to the mean home range of the species (male = 446 ha; female = 170 ha; Dunstone et al., 2002; Sanderson et al., 2002; Schüttler et al., 2017). Within each buffer area, which differed for males and females based on their average home ranges, we described or quantified five landscape variables: (i) percentage of vegetation cover (Hansen et al., 2013), (ii) number of houses, (iii) distance to the nearest house, (iv) land use (fragmented landscape or continuous forest) and (v) bioclimatic region: Mediterranean region, rainy‐temperate region, Chiloé Island (rainy‐temperate to oceanic cold‐temperate transition) region, and oceanic cold‐temperate region (INE, 2018). Land use (variable 4) is a qualitative variable with two categories. We defined continuous landscape as a buffer area composed only of continuous vegetation, which may include roads (as functional connectivity for guignas is not limited by roads; Gálvez et al., 2013, 2018; Sanderson et al., 2002). We defined fragmented landscape as a buffer area including human settlements, agricultural land and/or fragments of forest surrounded by a matrix of human activities. GIS layers were obtained from the Ministerio de Bienes Nacionales website. QGIS 2.14^®^ software was used to extract the attribute values of landscape variables for spatial analysis. We tested the collinearity among predictors by running bivariate Pearson correlations among pairs of variables. No statistically significant correlations were found. To address spatial autocorrelation in our data, we conducted a Global Moran's *I* test using ArcGIS Pro. We obtained non‐significant results (Moran's index = 0.38, *z*‐score = 0.46, *p*‐value = 0.64) suggesting there is no pattern of data spatial clustering.

### 2.5 Assessment of clinical signs of disease

We measured hematological, biochemical and histological parameters, and physical examination findings blind to viral PCR status. For hematological and biochemical analysis, whole blood preserved in EDTA of 20 guignas and serum samples of 19 guignas were tested. Hematological parameters analyzed included erythrocyte count (RBC), white blood cell count, hemoglobin concentration, mean cell volume, mean corpuscular hemoglobin concentration and hematocrit determination using the Abacus Junior Vet Analyzer (Diatron^®^). Biochemical parameters evaluated were glucose, total protein, albumin, globulin, total bilirubin, total cholesterol, blood urea nitrogen, creatinine, calcium, phosphorus, alanine aminotransferase, aspartate aminotransferase, and gamma glutamyltransferase, analyzed by Microlab 100 of MERCK^®^ employing Wiener® Lab chemical products.

For histopathological analysis, we collected samples during complete necropsies of 32 animals. Histological evaluation was performed on selected formalin‐fixed paraffin‐embedded tissues, sectioned at 5 μm and stained with hematoxylin eosin. Tissues were evaluated by a board‐certified pathologist blind to viral PCR status, geographic location, and other demographic information. Lesions were scored as present or absent and compared between PCR‐positive and PCR‐negative guignas. Immunohistochemistry using antibodies cross‐reactive to B (CD79a) and T (CD3) lymphocytes was performed following the manufacturer's protocol (Biocare Medical). Lymph node architecture was evaluated for number of follicles, presence and number of secondary follicles, lymphoid expansion into the paracortex, presence of lymphocytes within medullary cords and the presence of intrafollicular hyalinization, with results compared between positive and negative guignas.

### 2.6 Serological analysis

Serum samples from 16 live guignas were tested in serological analyses. Seven animals were tested by two different methods, the commercial ELISA kit INgezim FeLV DAS (Ingenasa) and SNAP FIV/FeLV Combo Test (IDEXX) to detect presence or absence of the p27 antigen. Five guignas were tested only by the IDDEX kit and four guignas were tested only with the INgezim kit. Only samples of sufficient quality were used for hematological, biochemical, histological and serological analyses. Tested animals included both PCR negative and PCR positive, to compare between infection statuses.

### 2.7 Statistical analysis

Spatial and biological (host age and sex) independent variables and FeLV and FIV infection (binary response variable) were assessed using multivariate logistic regression analyses, calculating crude and adjusted odds ratios (ORs) with 95% confidence intervals (CIs). Goodness of fit models were assessed using the Hosmer–Lemeshow test and residuals analysis. Differences in infection prevalence between domestic cat and guigna, and between biogeographic regions, as well as comparisons of hematological and biochemical parameters of infected and non‐infected guignas, were analyzed by Mann–Whitney *U* tests and Kruskal–Wallis tests. All statistical analyses were performed in R software with a significance level of *p* < 0.05.

## 3 RESULTS

### 3.1 Prevalence and genetic diversity

Feline leukemia virus‐infected guignas were detected in all bioclimatic areas (Table S2). FeLV‐infected domestic cats were detected in all rural communities studied across bioclimatic areas (Table S3). In contrast, FIV‐infected guignas were detected in only two of the four bioclimatic areas, while FIV‐infected domestic cats were identified in all bioclimatic areas (Tables S2,S3).

Feline leukemia virus DNA was detected in 20.6% of guignas and 20.2% of domestic cats, and FIV was detected in 3.0% of guignas and 3.0% of domestic cats. All FIV‐infected guignas were also coinfected with FeLV; FIV/FeLV co‐infections occurred in 1.14% of domestic cats (Tables S2,S3). There was no statistically significant difference in FeLV and FIV prevalence between the two host species (FeLV, *p* = 0.93, *U* = 13,310; FIV, *p* = 0.95, *U* = 13,350) or between years (*p* = 0.58).

Feline leukemia virus PCR products were sequenced (211 bp) from 13/21 PCR‐positive guignas and 25/53 PCR‐positive domestic cats, and FIV sequences from 2/3 PCR‐positive guignas and 5/8 PCR‐positive domestic cats. No amplification products were obtained for longer FeLV fragments (700 bp).

**FIGURE 2:**
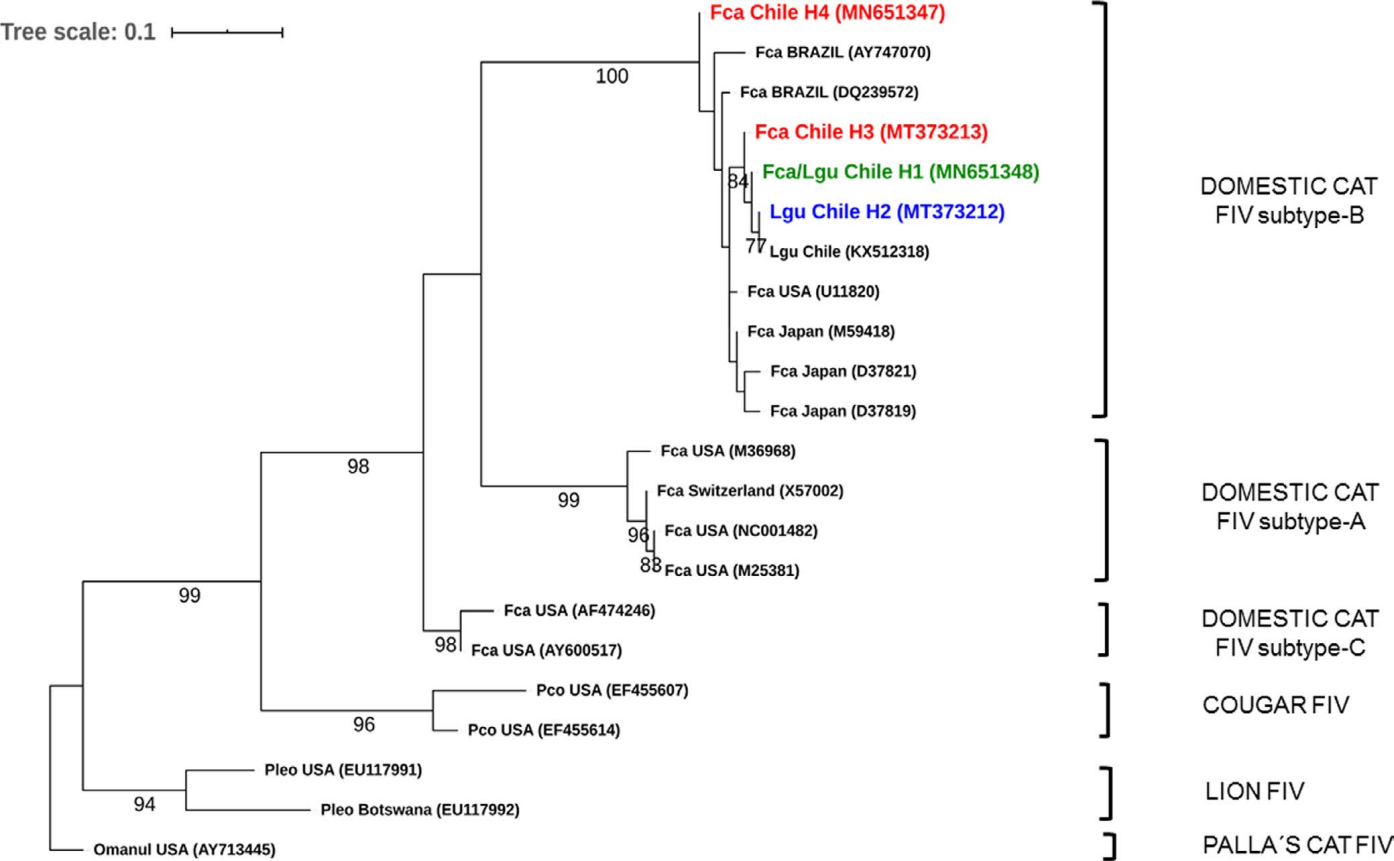
Maximum‐likelihood tree of 291 bp fragment of the FIV gag genomic region for guignas and domestic cats. Sequences from this study are highlighted (red = domestic cat NtST, green = domestic cat/guigna shared NtST, blue = guigna NtST). Numbers in sequence names correspond to the ntST described in this study (H1–H4). GenBank accession numbers in parentheses. Bootstrap values ≥70 are shown at the nodes of the tree. Lgu, *Leopardus guigna*; Fca, *Felis silvestris catus*; Pco, *Puma concolor*; Omanul, *Otocolobus manul*. FIV‐positive control = haplotype 4

**FIGURE 3:**
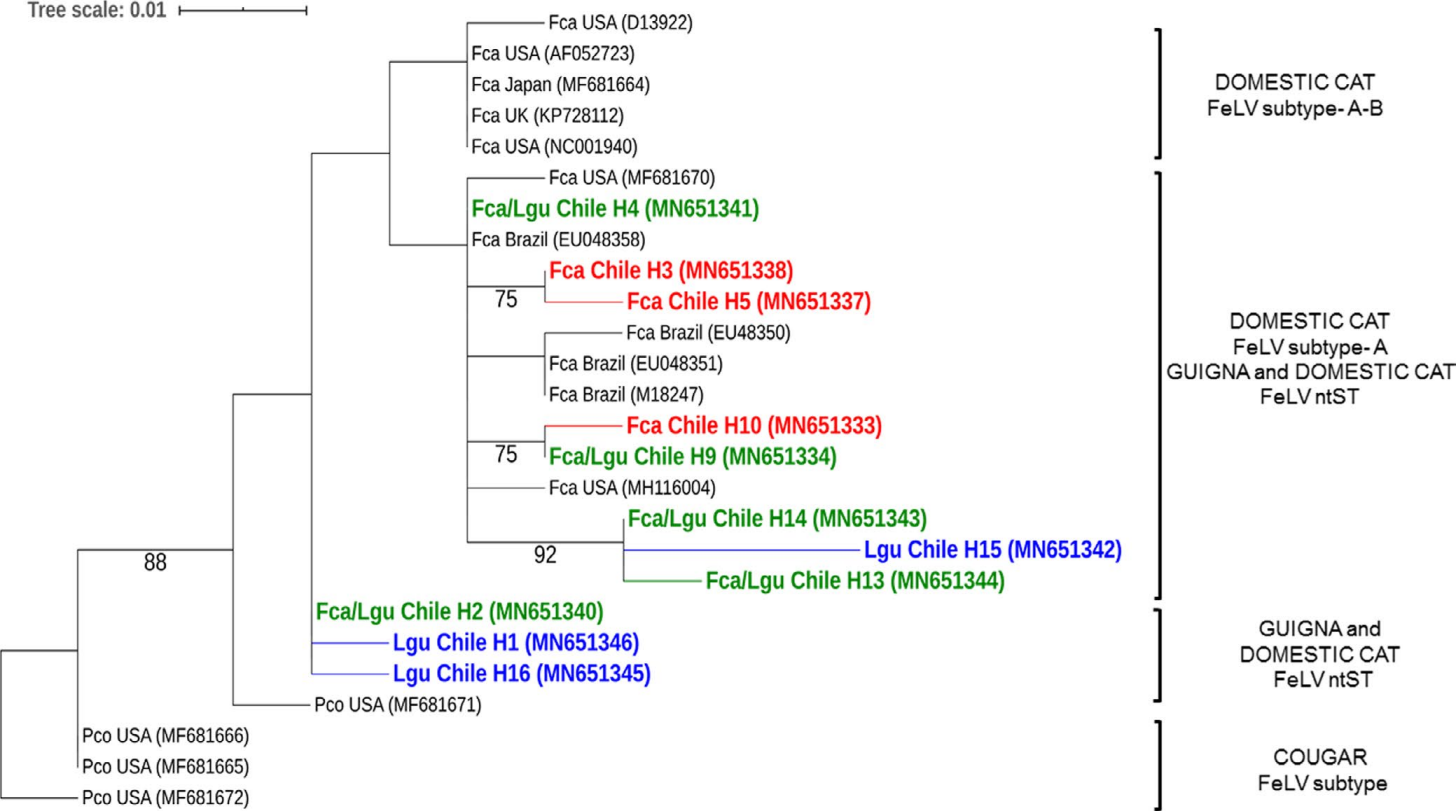
Maximum‐likelihood tree of 211 bp fragment of FeLV U3LTR genomic region for guignas and domestic cats. Sequences from this study are highlighted (red = domestic cat NtST, green = domestic cat/guigna shared NtST, blue = guigna NtST). Numbers in sequence names correspond to the ntST described in this study (H1, H2, H3, H4, H5, H9, H10, H13, H14, H15, H16). GenBank accession numbers in parentheses. Bootstrap values ≥70 are shown at the nodes of the tree. Lgu, *Leopardus guigna*; Fca, *Felis silvestris catus*; Pco, *Puma concolor*

Genetic analysis identified four FIV nucleotide sequence types (ntSTs), one of which was shared between the species (Figure 2). For FeLV, 16 ntSTs were identified, of which five (H2, H4, H9, H13, and H14) were shared between guignas and domestic cats (Figure 3). Five of these FeLV ntSTs were not included in the phylogenetic trees because the sequence fragments were shorter than the consensus GenBank alignment, but were sufficient to confirm the presence of proviral DNA and to assign viral haplotypes.

Bayesian and maximum‐likelihood FeLV and FIV phylogenetic trees had similar topologies and clustered domestic cat and guigna viral sequences together in well‐supported clades (Bayesian tree; Figures 2, 3 and S2). Guigna and domestic cat FIV sequences clustered together with domestic cat FIV subtype‐B sequences in a separate linage from those of other wild felid FIV sequences. Guigna and domestic cat FeLV sequences clustered together with FeLV sequences of domestic cats from North and South America. The FeLV phylogenetic tree showed a polytomy, probably due to the limited length of our sequence fragments.

Feline leukemia virus sequences had 98%–99% nucleotide similarity with GenBank FeLV‐A sequences of domestic cats from USA and 98% nucleotide similarity with FeLV‐B sequences of Florida panther from the USA. For FIV, guigna sequences had 98% similarity with FIV sequences of domestic cats from the USA.

The genetic distance between FeLV sequences from guignas and domestic cats of this study, and between guignas of the study and domestic cats worldwide (sequences obtained from GenBank, in phylogenetic tree), was 3%. The genetic distance between FIV sequences of guignas and domestic cats of this study was 1%, compared to wider differences between guignas and domestic cats worldwide (only FIV subtype B) of 4.7%.

NtST network analysis of FeLV and FIV sequences revealed no significant phylogeographic structure, suggesting that most of the ntSTs were widespread throughout the study area with no clear geographic clustering (Figure S1). This pattern is supported by the non‐significant results of the *G*_ST_‐*N*_ST_ tests (FeLV: *N*_ST_ = 0.012 ± 0; *G*_ST _= 0.009 ± 0.041, *p*‐value = 0.5; FIV: the *G*_ST_‐*N*_ST_ test could not be conducted due to the low number of sequences per location; Figure S1). Similarly, no phylogenetic structure was observed among FeLV and FIV sequences from guigna and domestic cat host species; haplotype frequencies (FeLV: Phi_st_ = 0.01, *p* = 0.32; FIV: Phi_st_ = 0.06, *p* = 0.45) and nucleotide sequence‐based statistics (FeLV: *S*_nn_ = 0.52, *p* = 0.64; FIV: *S*_nn_ = 0.57, *p* = 0.34) were not significantly different.

### 3.2 Exposure risk factor analysis

Guignas were significantly (9.6×) more likely to be exposed to FeLV in fragmented landscapes (95% CI = 2.37–65.32; *p* = 0.005; adjusted odds ratio = 9.6; Table S2) and male guignas were 3.6 times more likely to be infected with FeLV than females (95% CI = 1.18–12.42; *p* = 0.03; adjusted odds ratio=3.6; Hosmer–Lemeshow test for goodness of fit model, *p* = 0.99). No other statistically significant differences were observed for FeLV in guignas comparing ages, bioclimatic areas or for the other spatial variables, percentage of vegetation cover, number of houses, distance to the nearest house, or land use category (fragmented landscape or continuous forest). The three FIV‐positive guignas (also coinfected with FeLV), two adult males and one young female, were all found in fragmented landscapes. However, no statistical analysis for the spatial and biological variables studied was possible for FIV due to the low observed prevalence. A significantly lower FeLV prevalence was observed among domestic cats in the rainy‐temperate area (95% CI = 0.12–0.60; *p* = 0.01) and oceanic cold‐temperate area (95% CI = 0.14–0.81: *p* = 0.02). No statistically significant differences for domestic cat FeLV or FIV infection or coinfection were observed with respect to sex or age (95% CI = 0.55–1.91; *p* = 0.9 and 95% CI = 0.35–2.29; *p* = 0.9, respectively; Table S3).

### 3.3 Serology and clinical signs in guigna

Serum samples from four and seven FIV and FeLV PCR‐positive guignas, respectively, were analyzed and found negative using INgezim and IDEXX kits (Table 1). One FeLV‐infected adult male had gingivitis in the clinical evaluation. Only one individual, a FeLV‐infected young female, had hematological abnormalities compatible with FeLV infection, presenting anemia, leucopoenia, lymphopenia and neutropenia (established based on hematological reference values of *Leopardus geoffroyi*, the closest evolutionary species; Teare, 2002) and normal average hematological values of guignas not infected with FeLV or FIV; Table S4).

**TABLE 1:** Summary of serology and PCR FeLV results of guignas for which both analyses were conducted

ID	Sex	Age	ELISA (INgezim commercial kit)		ELISA (IDEXX commercial kit)		PCR	
			FeLV	FIV	FeLV	FIV	FeLV	FIV
LG001	Female	Juvenile	NA	NA	Negative	Negative	Positive	Positive
LG002	Male	Adult	Negative	Negative	Negative	Negative	Positive	Negative
LG003	Male	Adult	Negative	Negative	Negative	Negative	Negative	Negative
LG004	Male	Adult	Negative	Negative	Negative	Negative	Negative	Negative
LG005	Female	Adult	NA	NA	Negative	Negative	Positive	Negative
LG006	Male	Adult	Negative	Negative	Negative	Negative	Positive	Negative
LG007	Male	Adult	Negative	Negative	Negative	Negative	Positive	Negative
LG008	Female	Adult	Negative	Negative	Negative	Negative	Negative	Negative
LG009	Male	Adult	Negative	Negative	Negative	Negative	Positive	Negative
LG078	Male	Adult	NA	NA	Negative	Negative	Negative	Negative
LG080	Male	Adult	NA	NA	Negative	Negative	Positive	Positive
LG144	Male	Juvenile	Negative	Negative	NA	NA	Negative	Negative
LG159	Male	Juvenile	Negative	Negative	NA	NA	Negative	Negative
LG160	Male	Adult	Negative	Negative	NA	NA	Negative	Negative
LG153	Female	Adult	Negative	Negative	NA	NA	Negative	Negative
LG154	Female	Juvenile	Negative	Negative	NA	NA	Negative	Negative

Abbreviation: NA, Not analyzed.

There was no evidence of histopathological lesions compatible with FeLV or FIV disease associated with infection in positive or negative guignas. As a secondary finding, two adult males and one adult female had pulmonary anthracosis, all three belonging to anthropized areas. Lymph nodes from six guignas were available for morphometric evaluation. There was no difference in the total number of follicles, number of secondary follicles, paracortical or medullary cord lymphocytes among FeLV‐infected and uninfected guignas. To detect subtle alterations in B and T lymphocytes, lymph nodes were stained for CD79a (B cell) and CD3 (T cell) markers, however tissues failed to stain reliably, likely due to prolonged fixation in formalin.

## 4 DISCUSSION

The present study represents one of the largest surveys of retroviral infection in a wild feline and sympatric rural free‐roaming domestic cats. We surveyed guignas from across their complete distribution range in Chile, documenting for the first time that they are infected with FeLV and FIV throughout most of their range. Estimates of prevalence of FeLV and FIV for guignas and domestic cats were similar to those found in a previous pilot study on Chiloé Island (Mora et al., 2015). Previous records of retroviral prevalence in domestic cats in Chile ranged from 4%–15% for FIV and 4%–10% for FeLV (Bilbao, 2008; Troncoso et al., 2013).

All guigna viral sequences were most closely related phylogenetically to domestic cat viral strains from North and South America, Japan, and also UK (FeLV tree only), providing insights on the evolutionary origins of the FeLV and FIV strains infecting guignas in Chile and their potential transmission pathways. We acknowledge that with our data it is not possible to define unmistakably the direction of transmission. However, guigna viral sequences for both FeLV and FIV are clustered within clades composed of domestic cats from Chile, Brazil and USA (and also Japan for FIV and UK for FeLV), in contrast with what has been reported previously for FIV in several wild cat species where free‐ranging individuals harbor monophyletic, species‐specific strains (Pecon‐Slattery et al., 2008; Troyer et al., 2008). If guignas were infected with FeLV and FIV independently of domestic cat transmission or strains, guignas would have their own monophyletic species‐specific strains for both viruses, clustered in unique separate clades in the phylogenetic trees (in contrast to the results presented here).

Feline immunodeficiency virus is a species‐specific infection, and cross‐species transmission is highly restricted by interspecies transmission barriers. However, although rare, a few cross‐species transmissions have been reported: An FIV strain shared by both bobcats (*Lynx rufus*) and pumas (*Puma concolor*) occupying the same habitat in Florida and California (Franklin et al., 2007; Lee et al., 2014), a free‐ranging leopard cat that acquired FIVfca from a domestic cat (Nishimura et al., 1999), FIVfca in a captive puma in Argentina and lion FIV clade A sequences (FIVpleA) amplified from a snow leopard (*Panthera uncia*) and a tiger (*Panthera tigris*) in Asian zoos (Carpenter et al., 1996; Troyer et al., 2005).

Viruses isolated from different species seem to group more by geographic region of the host than in groupings concordant with the phylogenetic relationships of the host species (Troyer et al., 2008). Cross‐species transmission among bobcats and pumas in the USA is believed to have been facilitated by both species occupying the same habitat. A somewhat similar situation is apparent in the guigna and rural domestic cats sampled in this study, where animals may come into direct contact facilitated by guignas approaching households occasionally when preying on fowl (Sanderson et al., 2002), or domestic cats roaming freely up to 2 km away from the household into the forest (López‐Jara et al., unpublished data), increasing the probability that exposure to non‐native viruses would occur.

Haplotype networks showed several shared haplotypes between guignas and domestic cats, with no genetic structure in relation to host species, suggesting that these viruses are not species‐specific in guignas. The highest proportion of viral FeLV ntST was observed only in domestic cats (8/16), followed by shared ntST between both host species (5/16) including the central ntST, and finally the smallest proportion of ntST was only identified in guigna (3/16), all of which clustered in the network with few mutational steps of difference, suggesting new mutations within guigna populations after the virus had crossed the species barrier. A similar situation is observed for FIV ntST, although with many fewer data/haplotypes. Although we acknowledge that with these data it is not possible to define unmistakably the direction of transmission, overall this ntST network pattern suggests that the origin of viral ntST and infection was probably in domestic cats and not guignas. Our combined genetic evidence supports the existence of cross‐species transmission of FeLV and FIV between domestic cats and guignas.

Most FeLV sequences in guigna were closely related to the common horizontally transmissible FeLV‐A subgroup, originally amplified from domestic cats in the USA (GenBank MF681664). One guigna showed high nucleotide similarity with the FeLV‐B subgroup found in a Florida panther from USA (GenBank MF681671). This subgroup is a recombinant of FeLV‐A and enFeLV, an endogenous retrovirus only present in the genus *Felis* (Polani et al., 2010). The presence of FeLV‐B in guignas of the genus *Leopardus* is only possible though horizontal transmission from domestic cats, the only *Felis* species present in Chile.

Contrary to other studies that have identified species‐specific FIV infection in wild felids (VandeWoude & Apetrei, 2006), in guignas FIV sequences cluster together with the domestic cat FIV‐B subtype lineage, a worldwide widespread subtype in domestic cats (Burkhard & Dean, 2003), separated from strains of other wild felid species.

Guigna FeLV infection was also significantly associated with fragmented landscapes (nine times more probability of being infected), where humans and their domestic cats inhabit). Our combined evidence (genetic and spatial analysis) suggests domestic cats may be the most probable source of infection for guigna populations.

Domestic cats have been widely recorded as sources of FIV and FeLV infection in wild felids, in different species and contexts (e.g., domestic cat FeLV infection in Florida panthers, Chiu et al., 2019; O’Brien et al., 2012). Higher exposure to pathogens in wild carnivore populations and disease emergence have been reported in human‐dominated landscapes (Alexander & Appel, 1994; Cleaveland et al., 2000; Laurenson et al., 1998; Millán et al., 2016; Riley et al., 2004; Sillero‐Zubiri et al., 1996).

In Chilean fragmented landscapes with domestic cat presence, where guignas increase their dispersal (Napolitano, Díaz, et al., 2015) and occasional poultry attacks by guignas within human settlements occur, increased aggressive domestic cat‐guigna encounters may enhance opportunities for novel pathogen exposure and spillover (Mora et al., 2015; Sanderson et al., 2002). Considering that FIV and FeLV are shed in high concentrations in saliva and that the major mode of transmission is through bites (Sykes et al., 2014a, 2014b), these aggressive encounters may facilitate interspecific transmission. Once the species barrier has been crossed, it is possible for the virus to propagate among individuals of the new host species (Cunningham et al., 2008), followed by a level of interspecific encounters sufficient to allow virus transfer, establishment and adaptation (Engering et al., 2013; Parrish et al., 2008).

Male guignas had higher probability of FeLV infection than females; this is also the case for domestic cats due to more aggressive behavior (Hartmann, 2006; Munro et al., 2014). In most solitary mammals, females are philopatric and males disperse from their natal area, fiercely fighting for territory acquisition and access to females (Moyer et al., 2006; Prugnolle & De Meeûs, 2002; Ratnayeke et al., 2002; Waser & Jones, 1983). Although guignas are solitary felids, less likely to acquire an infectious agent through direct contact, male guignas are expected to play a role in intraspecific transmission of pathogens. Transmission of infection to females could follow from mating activities, when the tomcat bites the female (Meli et al., 2010).

Feline leukemia virus‐infected guignas and FeLV and FIV‐infected domestic cats were present in all bioclimatic areas studied. However, FIV‐infected guignas were detected in only two of these (rainy‐temperate region and Chiloé Island), perhaps because our more limited sampling in these bioclimatic areas was insufficient to detect the very low prevalence of FIV. Given that both pathogens are transmitted by direct contact, we would not expect bioclimatic variables to influence distribution and/or prevalence directly. However, higher rates observed in central Chile (Mediterranean area), where over 50% of Chile's population resides (Napolitano, Gálvez, et al., 2015), may be linked to higher domestic cat density (no official domestic cat census is available). Habitat changes in several parts of the rainy‐temperate region, including extensive and intensive native forest habitat loss and fragmentation are probably also contributing to higher occupancy of humans and domestic cats (Echeverria et al., 2008; Sanderson et al., 2002; Wilson et al., 2005).

This study represents the first comprehensive evaluation of whether FeLV and FIV are causing disease in guignas. We found a low frequency of clinical signs (compatible with FeLV infection, but not specific) and no histopathological evidence of disease in infected guignas. However, our sample sizes were relatively small, and many cats died due to other acute causes such as vehicular trauma, precluding normal infection and disease course. Proviral DNA detected by molecular tests is integrated in the genome and could potentially reactivate and/or recombine with other viral subtypes, leading to emerging diseases and posing future threats for guignas, including potential population extinctions and impacting the species' long‐term viability.

Feline immunodeficiency virus infection is usually endemic (Troyer et al., 2005). Because of a long history of co‐evolution, felids are generally well adapted to their presence if ecosystems are stable and they develop immunity, so disease is not usually observed (Carpenter & O’Brien, 1995; Olmsted et al., 1992; Packer et al., 1999; Troyer et al., 2005). Evidence of morbidity and mortality caused by FIV infection have rarely been documented in free‐ranging wild felids; it does not appear to have an immunosuppressive effect (Pedersen & Barlough, 1991; Roelke et al., 2009) or typically affects only individual animals rather than populations (Troyer et al., 2005).

In contrast, FeLV does not appear to be endemic for most free‐ranging populations of wild felines, except for European wildcats (*Felis silvestris*; Millán et al., 2009). Therefore, it is likely that most free‐ranging felid species have not developed resistance to this virus and may develop fatal disease due to its immunosuppressive and carcinogenic effects (Cunningham et al., 2008; Marker et al., 2003). FeLV epidemics have occurred in wild felids, with virulent behavior, relatively rapid clinical course (in comparison to domestic cats) and causing significant mortality in all infected individuals (Florida panther, Cunningham et al., 2008; captive cheetah (*Acinonyx jubatus*), Marker et al., 2003; Iberian lynx, Meli et al., 2010).

All FeLV PCR‐positive guignas analyzed in this study were discordant to serological testing. The results should be interpreted with caution, since serologic tests were designed for domestic cat use and have not been validated for guigna, so there may be no cross‐reactivity, yielding false negative results. However, for antigen tests there should be no difference in testing guigna or domestic cat. In fact, Franklin et al. (2007) found the commercial ELISA kit IDDEX may provide an adequate alternative for FIV screening of bobcats, pumas, and ocelots (*Leopardus pardalis*). Thus this discordancy between PCR and ELISA likely suggests that at least some of the guignas are regressively infected. Alternatively, the antibody may have been degraded.

Despite several attempts we were unable to amplify longer viral fragments, therefore it is likely that viral loads in infected guignas are too low to be detected by these methods. FeLV PCR‐positive guignas are probably regressively infected, usually having no clinical signs as we report here, being non‐viremic, not shedding virus (thus not horizontally infectious, but vertical infection does occur) and serology negative (Hartmann, 2012). Regressive infection could potentially reactivate into progressive infection (Hartmann, 2012). However, with our limited sampling we cannot calculate the proportion of regressive versus progressive infection (if any) in guignas. Future studies should aim to sequence whole viral genomes to provide more complete evidence.

Predicting and preventing epidemics in wild felids will require a comprehensive understanding of the ecology and pathogenesis of infectious diseases. Only with continuous research and monitoring, accurate diagnostic tools and strong predictive models will we be able to prevent disease from emerging as a significant factor in wild felid survival. Managers and national authorities should consider limiting access of domestic dogs and cats to natural areas in combination with the expansion of disease management programs throughout Chile, including vaccination programs (as are already being implemented by the Ministry of the Environment in some areas), responsible pet ownership promotion, increased law enforcement of existing regulations and by facilitating veterinary care in rural landscapes. This study is the first comprehensive assessment of FeLV and FIV infection in guignas from across their entire distribution in Chile, enhancing our knowledge on pathogen transmission at the wildlife–domestic interface.

## CONFLICT OF INTEREST

The authors declare no competing interests. Parts of this manuscript were prepared while Warren E. Johnson held a National Research Council Research Associateship Award at the Walter Reed Army Institute of Research (WRAIR). The material has been reviewed by WRAIR, and there is no objection to its presentation and/or publication. The opinions and assertions contained herein are the private views of the authors and are not to be construed as official, or as reflecting true views of the Department of the Army or the Department of Defense.

## Supporting information

Supplementary Material

Fig S1

Fig S2

## Data Availability

Derived data supporting the findings of this study are available from the corresponding author upon request.
