# Supplementary material for: Cross‐species transmission of retroviruses among domestic and wild felids in human‐occupied landscapes in Chile
Source: Evol Appl. 2021 Jan 27;14(4):1070–82. doi: 10.1111/eva.13181 (PMC8061269; doi:10.1111/eva.13181)
Supplement: Supplementary file 1 — Supplementary Material [file EVA-14-1070-s002.docx]

**Supplementary Material**

**Tables.**

**Table S1.** Newly identified FeLV and FIV sequences submitted to the GenBank database under listed accession numbers.

| FeLV sequences | Genbank accession number |
| --- | --- |
| Nucleotide sequence type H1 | MN651346 |
| Nucleotide sequence type H2 | MN651340 |
| Nucleotide sequence type H3 | MN651338 |
| Nucleotide sequence type H4 | MN651341 |
| Nucleotide sequence type H5 | MN651337 |
| Nucleotide sequence type H6 | MN651331 |
| Nucleotide sequence type H7 | MN651336 |
| Nucleotide sequence type H8 | N651335 |
| Nucleotide sequence type H9 | MN651334 |
| Nucleotide sequence type H10 | MN651333 |
| Nucleotide sequence type H11 | MN651332 |
| Nucleotide sequence type H12 | MN651339 |
| Nucleotide sequence type H13 | MN651344 |
| Nucleotide sequence type H14 | MN651343 |
| Nucleotide sequence type H15 | MN651342 |
| Nucleotide sequence type H16 | MN651345 |
| FIV sequences |  |
| Nucleotide sequence type H1 | MN651348 |
| Nucleotide sequence type H2 | MT373212 |
| Nucleotide sequence type H3 | MT373213 |
| Nucleotide sequence type H4 | MN651347 |

**Table S2.** Observed prevalence of feline leukemia virus (FeLV), feline immunodeficiency virus (FIV) infection and FeLV-FIV coinfection in guigna according to landscape composition, sex, age and bioclimatic regions, across guigna distribution in Chile.

| Pathogen | % Positive  (CI 95%)  Total | % Positive  (CI 95%)  Fragmented landscape | % Positive  (CI 95%)  Continuous Forest | % Positive  (CI 95%)  Male | % Positive  (CI 95%)  Female | % Positive  (CI 95%)  Adult | % Positive  (CI 95%)  Juvenile | % Positive  (CI 95%)  Mediterranean  area | % Positive  (CI 95%)  Rainy-temperate area | % Positive  (CI 95%)  Chiloé Island | % Positive  (CI 95%)  Oceanic-cold temperate area |
| --- | --- | --- | --- | --- | --- | --- | --- | --- | --- | --- | --- |
|  | *n*=102 | *n*=69 | *n*=33 | *n*= 64 | *n*=38 | *n*= 63 | *n*=16 | *n*=27 | *n*=18 | *n*=36 | *n*=21 |
| FeLV | 20.6  (12.6-28.6) | 27.54  (16.7-38.35) | 6.0  (-2.5-14.6) | 25.0  (14.1-35.9) | 13.2  (1.9-24.4) | 25.4  (14.3-36.4) | 18.7  (-2.7-40.2) | 29.6  (11.2-48.0) | 11.1  (-5.0-27.2) | 25.0  (10.1-39-9) | 9.5  (-0.04-23.2) |
| FIV | 3.0  (0.3-6.2) | 5.3  (0.7-11.4) | 0.0  (0.0-0.0) | 2.0  (0.7-4.7) | 1.0  (-0.9-2.9) | 2.0  (0.7-4.7) | 2.0  (0.7-4.7) | 0.0  (0.0-0.0) | 0.98  (-0.9-2.9) | 2.0  (0.7-4.7) | 0.0  (0.0-0.0) |
| FeLV-FIV coinfection | 3.0  (0.3-6.2) | 4.3  (-0.5-9.2) | 0.0  (0.0-0.0) | 2.0  (0.7-4.7) | 0.98  (-0.9-2.9) | 1.96  (0.7-4.7) | 1.96  (0.7-4.7) | 0.0  (0.0-0.0) | 0.98  (-0.9-2.9) | 1.96  (0.7-4.7) | 0.0  (0.0-0.0) |

**Table S3.** Observed prevalence of FeLV and FIV infection in domestic cats by sex, age and bioclimatic area among guignas distribution in Chile.

|  | Overall | Sex | | Age | | Bioclimatic area | | | |
| --- | --- | --- | --- | --- | --- | --- | --- | --- | --- |
| Pathogen | % Positive  (CI 95%)  Total  *n*=262 | % Positive  (CI 95%)  Male  *n*=133 | % Positive  (CI 95%)  Female  *n*=129 | % Positive  (CI 95%)  Adult  *n*=226 | % Positive  (CI 95%)  Juvenile  *n*=36 | % Positive  (CI 95%)  Mediterranean area  *n*=43 | % Positive  (CI 95%)  Rainy-temperate-area  *n*=88 | % Positive  (CI 95%)  Chiloe Island  *n*=71 | % Positive  (CI 95%)  Oceanic-cold temperate area  *n*=60 |
| FeLV | 20.2  (15.3-25.1) | 20.3  (13.4-27.2) | 20.2  (13.1-27.2) | 20.3  (15.1-25.6) | 19.4  (5.9-33.0) | 20.9  (8.3-33.6) | 12.5  (5.4-19.5) | 33.8  (22.5-45.1) | 15.0  (5.7-24.3) |
| FIV | 3.0  (0.9-5.1) | 3.0  (0.1-5.9) | 3.1  (0.1-6.1) | 3.5  (1.1-6) | 0.0  (0.0-0.0) | 2.3  (-0.02-7.0) | 4.5  (0.1-9.0) | 2.8  (-1.1-6.8) | 1.7  (-1.7-5.0) |
| FeLV-FIV  coinfection | 1.1  (-0.1-2.4) | 0.7  -0.7-2.2 | 1.5  -0.6-3.1 | 1.3  -0.2-2.8 | 0.0  (0.0-0.0) | 2.3  (-2.3-7.0) | 1.1  (-1.1-3.4) | 1.5  (-1.4-4.2) | 0.0  (0.0-0.0) |

**Table S4.** Hematological parameters of FeLV-positive guigna compared with hematological values (mean, minimum and maximum) of FeLV noninfected guignas and hematological normal values of *Leopardus geoffroyi,* the most-directly related species with the guigna.

| **ID** | **Sex** | **Age** | **FeLV**  **status** | **FIV**  **status** | **RBC (x10^6^/µL)** | **Hemoglobin**  **(g/dL)** | **Hematocrit**  **(%)** | **MCV**  **fL** | **MCHC**  **g/dL** | **WBC**  **(x10^3^/ μL^-1^)** | **Segmented neutrophil**  **(x10^3^/ μL^-1^)** | **Lymphocyte**  **(x10^3^/ μL^-1^)** | **Monocyte**  **(x10^3^/ μL^-1^)** | **Eosinophil**  **(x10^3^/ μL^-1^)** |
| --- | --- | --- | --- | --- | --- | --- | --- | --- | --- | --- | --- | --- | --- | --- |
| ***Leopardus geoffroyi**** | **_** | **_** | **_** | **_** | **6.71-9.25** | **11.5-14.9** | **35.2-47.8** | **47-55.8** | **30.2-35.6** | **5.38-14.22** | **3.35-9.16** | **1.03-3.15** | **0.06-0.56** | **0-1.44** |
| Median (min.-max.)  FeLV negative guignas |  |  |  |  | 7.4  (5.8-9.3) | 13.0  (10.0-15.9) | 40.99  (33.0-41.0) | 55.4  (46.0-65.0) | 31.7  (27.0-39.8) | 10.8  (3.8-21.7) | 7.6  (2.0-15.4) | 3.0  (0.8-11.4) | 0.36  (0.1.1.3) | 0.13  (0.0-0.6) |
| LG163 | Female | Juvenile | (+) | (-) | 1.1** | 18.9 | 60.0** | 54.0 | 35.0** | 5.2** | 4.6** | 0.364** | 0.156 | 0.00 |

**Abnormal (outlier) hematological values in relation to the normal values of *Leopardus geoffroyi.*

**Supplementary Material**

**Figures.**

**Figure S1.** NtST network of guigna and domestic cat FeLV and FIV sequences. Each circle in the network corresponds to a different haplotype, the size of the circles corresponds to haplotype frequencies, the color of the circles corresponds to the four different bioclimatic areas and two host species (L. guigna and domestic cat). FeLV positive control = haplotype 6.

**Figure S2.** Bayesian tree of 211 bp fragment of FeLV U3LTR genomic region for guignas and domestic cats. Sequences from this study are highlighted (red=domestic cat NtST, green=domestic cat/guigna shared NtST, blue=guigna NtST). Numbers in sequences’ names correspond to the ntST described in this study (H1, H2, H3, H4, H5, H9, H10, H13, H14, H15, H16). GenBank accession numbers between parentheses. Posterior probability values are displayed at the nodes of the tree. Lgu= *Leopardus guigna*, Fca= *Felis silvestris catus*, Pco= *Puma concolor*.
