# Supplementary figures and images for: Cross‐species transmission of retroviruses among domestic and wild felids in human‐occupied landscapes in Chile
Source: Evol Appl. 2021 Jan 27;14(4):1070–82. doi: 10.1111/eva.13181 (PMC8061269; doi:10.1111/eva.13181)

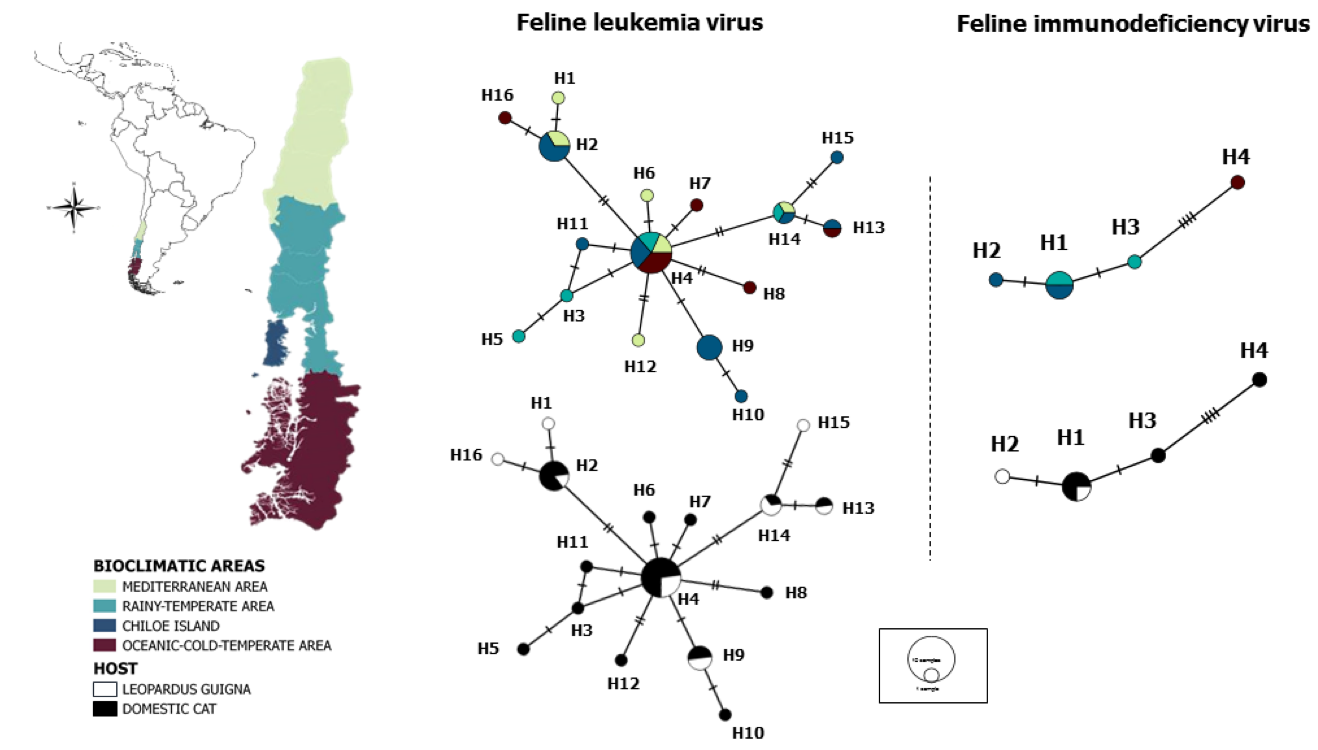

Supplement: Supplementary file 2 — Fig S1 [file EVA-14-1070-s001.png]

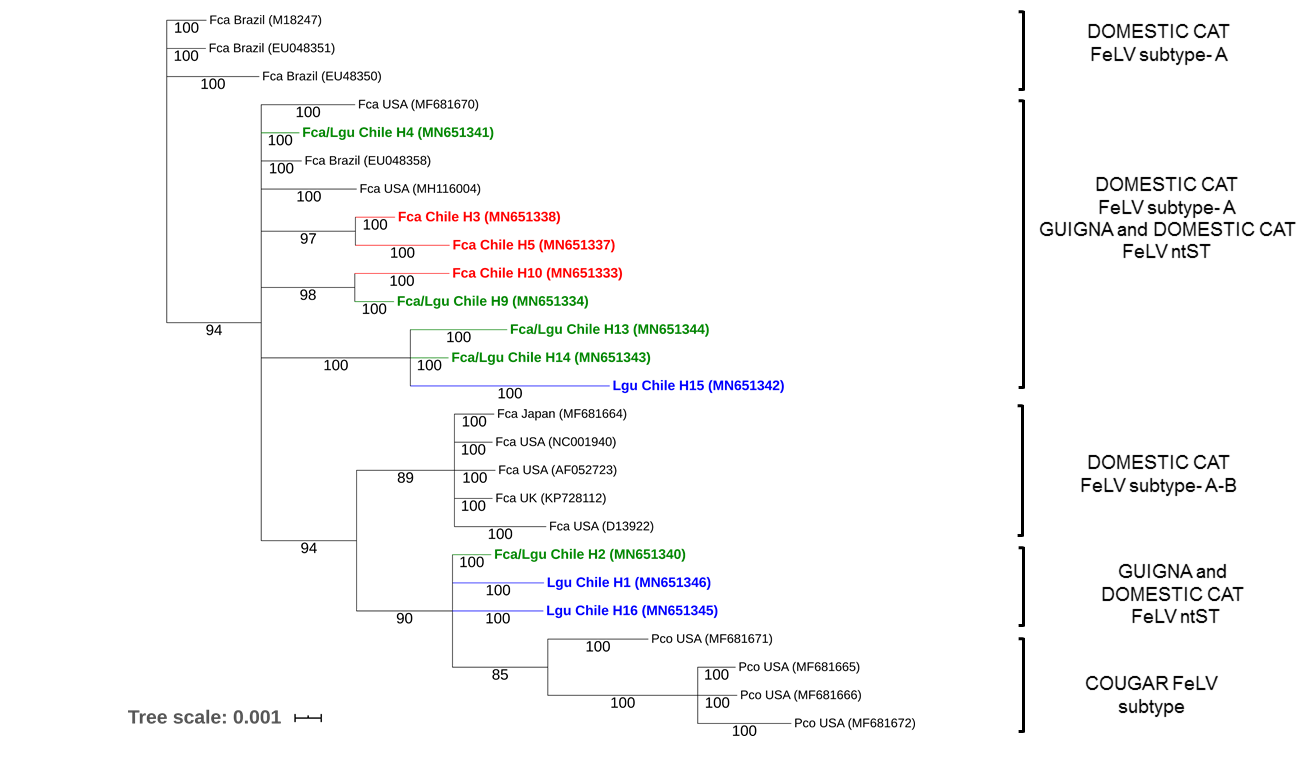

Supplement: Supplementary file 3 — Fig S2 [file EVA-14-1070-s003.tif]
